# Transcriptomic analysis reveals flavonoid biosynthesis of *Syringa oblata* Lindl. in response to different light intensity

**DOI:** 10.1186/s12870-019-2100-8

**Published:** 2019-11-11

**Authors:** Yan-Yan Liu, Xing-Ru Chen, Jin-Peng Wang, Wen-Qiang Cui, Xiao-Xu Xing, Xue-Ying Chen, Wen-Ya Ding, Bello-Onaghise God’spower, Nsabimana Eliphaz, Meng-Qing Sun, Yan-Hua Li

**Affiliations:** 1 College of Veterinary Medicine, Northeast Agricultural University, 600 Changjiang Road, Xiangfang, Harbin, Heilongjiang 150030 People’s Republic of China; 2 Heilongjiang Key Laboratory for Animal Disease Control and Pharmaceutical Development, Harbin, China

**Keywords:** *Syringa oblata* Lindl., Rutin, Flavonoid biosynthetic pathway, Light intensity, Metabolite, Transcriptome

## Abstract

**Background:**

Hazy weather significantly increase air pollution and affect light intensity which may also affect medicinal plants growth. *Syringa oblata* Lindl. (*S. oblata*), an effective anti-biofilm medicinal plants, is also vulnerable to changes in plant photoperiods and other abiotic stress responses. Rutin, one of the flavonoids, is the main bioactive ingredient in *S. oblata* that inhibits *Streptococcus suis* biofilm formation. Thus, the present study aims to explore the biosynthesis and molecular basis of flavonoids in *S. oblata* in response to different light intensity.

**Results:**

In this study, it was shown that compared with natural (Z_0_) and 25% ~ 35% (Z_2_) light intensities, the rutin content of *S. oblata* under 50% ~ 60% (Z_1_) light intensity increased significantly. In addition, an integrated analysis of metabolome and transcriptome was performed using light intensity stress conditions from two kinds of light intensities which *S. oblata* was subjected to: Z_0_ and Z_1_. The results revealed that differential metabolites and genes were mainly related to the flavonoid biosynthetic pathway. We found out that 13 putative structural genes and a transcription factor *bHLH* were significantly up-regulated in Z_1_. Among them, integration analysis showed that 3 putative structural genes including *4CL1*, *CYP73A* and *CYP75B1* significantly up-regulated the rutin biosynthesis*,* suggesting that these putative genes may be involved in regulating the flavonoid biosynthetic pathway, thereby making them key target genes in the whole metabolic process.

**Conclusions:**

The present study provided helpful information to search for the novel putative genes that are potential targets for *S. oblata* in response to light intensity.

## Background

Hazy weather is a normal event in China [1] that can severely increase the occurrence of air pollution [2]. It is characterized by scattering of particles which constitute the main factor for visibility degradation [3, 4]. On a longer timescale, these pollutants from China may affect North America, the Pacific and the Arctic, making Chinese air pollution a global problem [5]. Its direct result is that plants receive less light than in better weather condition. However, the plants in their natural habitats need to modify their growth and development to suit various environmental conditions including light signals [6], cold hardiness, drought resistance and shade abilities [7]. High light intensity [8] and UV-B radiation [9] also play an important role in the regulation of the flavonoids biosynthesis which is one of the most intensively studied area [10, 11]. It is well-known that flavonoids are the main secondary metabolites in medicinal plants in nature and have various biological functions, such as flower pigmentation, pollen fertility, plant microbe interaction, protection from UV radiation [12], antioxidant functions [13], anti-fungal and anti-bacterial properties [14]. Rutin is one of the well-known flavonoids in plants and has been reported as the main bioactive ingredient in *S. oblata* that inhibits *Streptococcus suis* (*S. suis*) biofilm formation [15]. Thus, it is unknown whether the concentration of flavonoids in *S. oblata* is affected by different light intensity groups and the effect of secondary metabolites against the formation of biofilm by *Staphylococcus xylosus* (*S. xylosus*) remains unexplored.

Recently, metabolomics analysis and transcriptomics [16, 17] have been successfully applied as connection networks in identifying differential gene expression in several plants, including *Yukon thellungiella* [18], sunflower [19] and *Nicotiana tabacum* [20]. However, no studies on bio-information analysis and functional identification have been published exploring the molecular mechanism of the secondary metabolite of flavonoid biosynthesis in the absence of *S. oblata* genomic information. Transcriptome sequencing [21, 22] is a rapid technique for obtaining functional genomic information that is widely used to determine gene structures and expression profiles in medicinal plants. Nevertheless, de novo assembly of RNA-Seq data makes it possible to conduct gene analysis in the absence of reference genomes [23, 24].

In this study, biofilm formation and flavonoids biosynthesis were examined under different light intensity groups in *S. oblata.* First, the changes in rutin content and the effect of *S. oblata* against the formation of biofilm by *S. xylosus* were tested in vitro in different months and different light intensity groups. Then, under the different light intensity groups, the presence of flavonoids in *S. oblata* were detected using histochemical method. Finally, comparative metabolomic and transcriptomic analysis were performed to identify the differentially expressed metabolites and genes. To the best of our knowledge, the study offers a new approach to the use of multi-omics technology for high-throughput sequencing in elucidating the molecular mechanism underlying the changes in the rutin content and flavonoid accumulation in *S. oblata* under different light intensity groups.

## Results

### Biofilm formation ability of *S. xylosus*

**Fig. 1:**
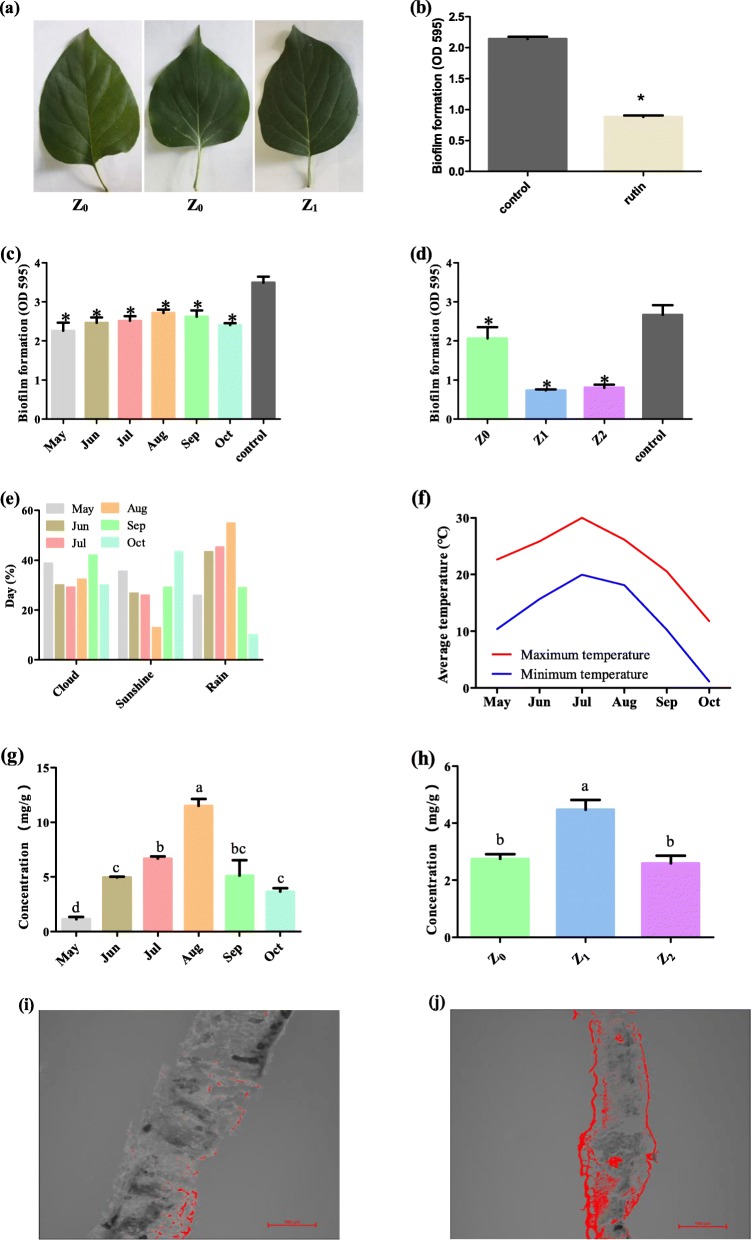
The leaves of *Syringa oblata* Lindl. grow under different light intensities such as natural (Z_0_), 50% ~ 60% (Z_1_) and 25% ~ 35% (Z_2_) light intensity (**a**). The inhibition of *S. xylosus* ATCC 700404 biofilm formation by rutin (**b**) the biological activity of *Syringa oblata* Lindl. against the formation of biofilm by *S. xylosus* ATCC 700404 during the different months (**c**) and light intensity (**d**) groups. The control (**b**, **c** and **d**) is *S. xylosus* ATCC 700404. Z_0_, Z_1_ and Z_2_ were natural, 50% ~ 60 and 25% ~ 35% light intensity, respectively. *p < 0.05 represents significantly different treatment means compared to untreated control bacterial culture. Changes in weather (**e**) and average temperature change (**f**) in different months in 2017. The rutin content of *Syringa oblata* Lindl. with different months (**g**) and light intensity (**h**) groups. Flavonoid accumulation in *Syringa oblata* Lindl. leaf cross sections detected by DPBA fluorescence (red) in natural (Z_0_) and 50% ~ 60% (Z_1_) light intensity in (**i**) and (**j**)

**Fig. 2:**
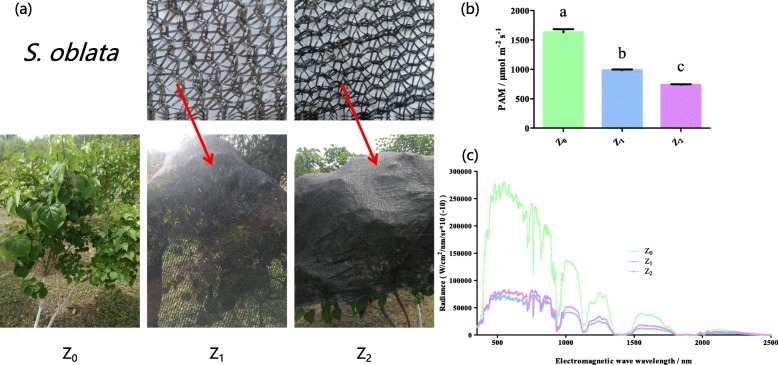
Different light intensity treatment design of *Syringa oblata* Lindl. in natural (Z_0_), 50% ~ 60% (Z_1_) with a black net and 25% ~ 35% (Z_2_) light intensity with a black net (**a**). (**b**) and (**c**) represent the photosynthetically active radiation (PAR) and light reflection of *Syringa oblata* Lindl. under different treatments in July in 2017, respectively

The study involves the evaluation of the biofilm inhibitory properties of rutin and *S. oblata* extract against *S. xylosus* biofilm formation during the period of different months and light intensity groups (Fig. 1a and Fig. 2a). The results revealed that 0.8 mg/mL of rutin significantly inhibited biofilm formation compared with the control (*p* < 0.05) (Fig. 1b). And the results showed that the MICs of all the different months of *S. oblata* against *S. xylosus* was 62.5 mg/mL, and 1/2 MIC (31.25 mg/mL) of the different months was able to significantly inhibit biofilm formation compared with the positive control (*p* < 0.05) (Fig. 1c). Furthermore, the MICs of Z_0_, Z_1_ and Z_2_ of *S. oblata* against *S. xylosus* were 62.5 mg/mL, 31.25 mg/mL and 31.25 mg/mL, respectively. Among them, the results revealed that in comparison with positive control, 1/2 MIC (15.625 mg/mL) of Z_1_ and Z_2_ significantly inhibited *S. xylosus* biofilm formation as against 1/2 MIC (31.25 mg/mL) of Z_0_ (*p* < 0.05) (Fig. 1d). This indicated that *S. oblata* had the highest ability to inhibit the formation of *S. xylosus* biofilm only when subjected to light intensity.

### Variations in the rutin content

In order to evaluate whether the content of rutin was influenced by the 6 months (May to October) and three light intensity groups (Z_0_, Z_1_ and Z_2_), two experiments were conducted. The trends of the daily sunshine, rainfall, and atmospheric temperature all through the months in the first experiment are shown in Fig. 1e, f. It revealed that the rutin content in May was 1.3331 ± 0.5612 mg/g, and then it increased to the highest value (11.0787 ± 0.9570 mg/g) in August. Then, it decreased in September (5.0921 ± 1.8441 mg/g) and October (3.6752 ± 0.7840 mg/g) (Fig. 1g). At the same time, the trend of the daily sunshine and rain fall all through the months were negatively and positively correlated with the content of rutin, respectively (Fig. 1g). The air pollution indices in August and September were more excellent than those obtained in other months, the values were obtained from the Website (https://www.aqistudy.cn/historydata/) (Additional file 1: Table S1). At the same time, lower particles such as ambient particulate matter (PM 2.5 and PM 10) could enhance solar radiation, thus enhancing ambient temperature and biological plant growth rates [25]. This finding is in agreement with previously findings reported on the content of rutin in Fructus Sophorae which peaked in mid-August, then declined gradually [26]. Furthermore, in the second experiment, the photosynthetically active radiation (PAR) and light reflection values of Z_1_ and Z_2_ measured by spectrometer were significantly lower than Z_0_ (Fig. 2b, c). The study revealed that the amount of rutin in *S. oblata* in September significantly increased in Z_1_ (4.4729 ± 0.7738 mg/g) when compared with Z_0_ (2.7518 ± 0.2854 mg/g) and Z_2_ (2.5921 ± 0.5419 mg/g) (*p* < 0.05) (Fig. 1h). Due to insufficient exposure of the leaves to light, the flavonoid content decreased after excessive shading which is also the reason why the rutin content in Z_2_ was lower than Z_1_. Therefore, Z_0_ and Z_1_ of *S. oblata* were selected and used for the next phase of studies [27].

### Histochemical analysis of flavonoids with different light intensity groups

Flavonoid accumulation in plants can be visualized using DPBA, a reagent, which indicates the presence of many flavonoids in histochemical analysis [28]. Leaves were stained with DPBA. Cross-sections of green fluorescence were observed in *S. oblata* (Fig. 1i, j) using LCSM. This finding is in agreement with earlier findings previously reported [28] that the presence of flavonoids was confirmed in different light intensity groups, especially observable in the epidermal cell layers and vascular bundles [29, 30]. To visualize the accumulation of flavonoids in leaf tissue more quantitatively and precisely, DPBA imagery was used. The results showed that the green fluorescence area of Z_1_ (2707423) was also larger than Z_0_ (903,872.5) after handling them with ImageJ (https://imagej.nih.gov/ij/index.html) [31].

### Metabolites analysis with different light intensity groups

**Fig. 3:**
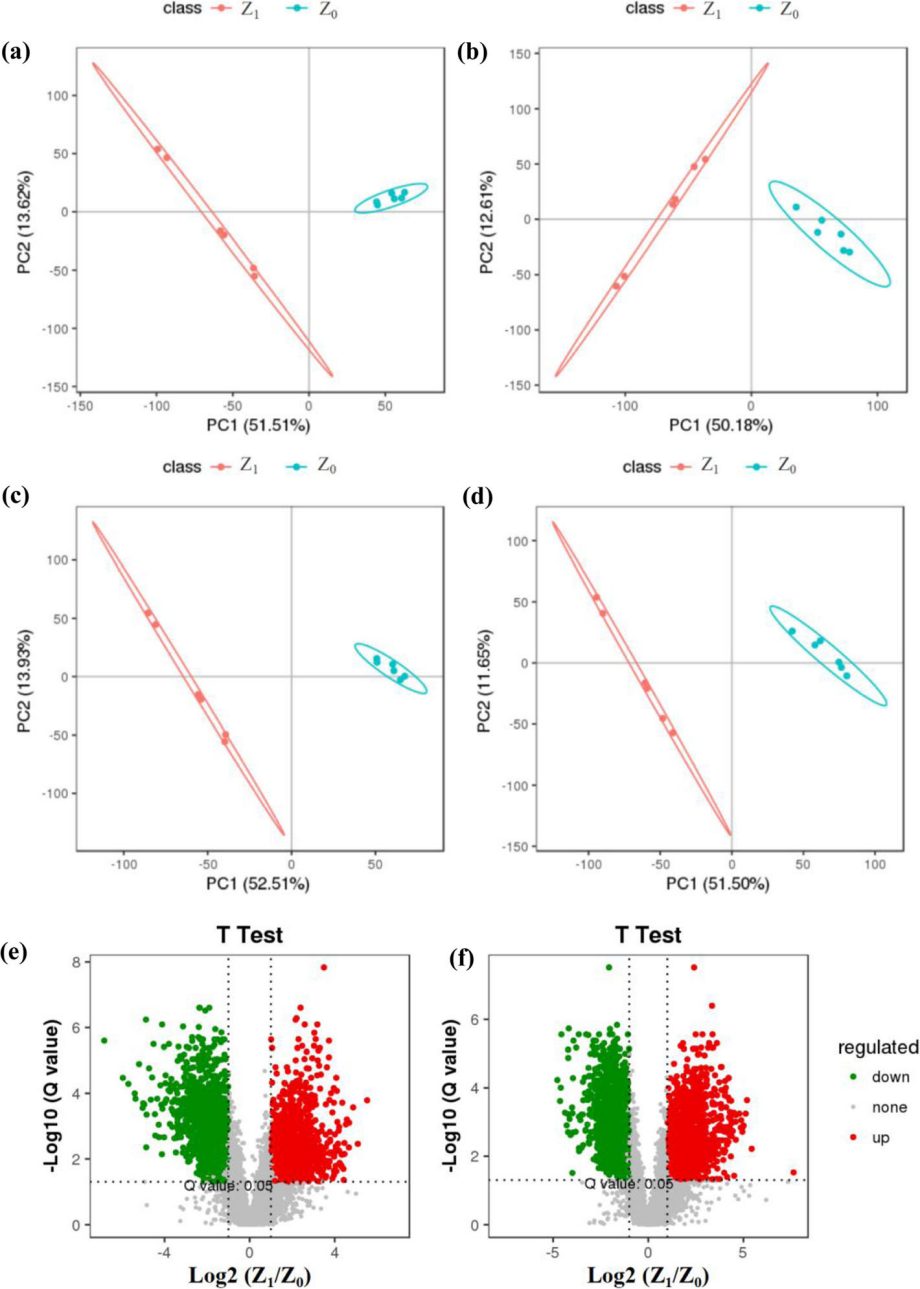
PCA, PLS-DA and Volcano of natural (Z_0_) and 50% ~ 60% (Z_1_) light intensity treatment of *Syringa oblata* Lindl. in the negative (**a**, **c**, **e**) and positive (**b**, **d**, **f**) ion modes

To identify key metabolic alteration after different light intensity groups, the metabolite levels between Z_0_ and Z_1_ were compared using LC-MS analysis. All data on retention time, exact mass, and peak intensity were recorded for multiple statistical analysis, including principal component analysis (PCA) (Fig. 3a, b) and partial least squares-discriminate analysis (PLS-DA) (Fig. 3c, d). These analytical methods revealed a trajectory of the different light intensity groups by the combination of the two main components. Volcano plot can visually screen for differentially expressed metabolites of *S. oblata* between the different light intensity groups. All metabolites from secondary mass spectrometry identification statistics were significantly different by ratio ≥ 2 or ratio ≤ 1/2, *q*-value≤0.05 and VIP ≥ 1 in different ion modes. The result showed that there were 7402 and 9481 total metabolites in the negative and positive ions modes contained as (1212, 1553) up-regulated and (1439, 1749) down-regulated metabolites, respectively (Fig. 3e, f). Furthermore, the secondary metabolite matching to specific biosynthesis pathways in terms of the number of matches in the positive and negative ions modes were enriched and analyzed by the KEGG pathway. The results showed that the secondary metabolite biosynthesis pathways were mainly matched to isoquinoline alkaloid biosynthesis, glucosinolate biosynthesis, and flavonoid biosynthesis (Table 1).

**Table 1:** KEGG pathway analysis on biosynthesis of other secondary metabolites

Pathway	Number of Compound (Detected)			Pathway ID
	All	Negative	Positive	
Isoquinoline alkaloid biosynthesis	93	37	64	map00950
Glucosinolate biosynthesis	75	19	28	map00966
Flavonoid biosynthesis	68	52	43	map00941
Tropane, piperidine and pyridine alkaloid biosynthesis	68	8	30	map00960
Phenylpropanoid biosynthesis	66	33	47	map00940
Anthocyanin biosynthesis	66	20	44	map00942
Isoflavonoid biosynthesis	63	52	44	map00943
Flavone and flavonol biosynthesis	49	38	35	map00944
Indole alkaloid biosynthesis	47	25	28	map00901
Monobactam biosynthesis	39	21	22	map00261
Carbapenem biosynthesis	32	11	11	map00332
Stilbenoid, diarylheptanoid and gingerol biosynthesis	25	12	16	map00945
Betalain biosynthesis	24	10	15	map00965
Caffeine metabolism	21	11	7	map00232
Benzoxazinoid biosynthesis	9	3	4	map00402
Acridone alkaloid biosynthesis	7	4	5	map001058

### Transcriptome sequencing and data analysis with different light intensity groups

Transcriptome sequencing results and sequence assembly.

In order to understand the potential molecular synthesis mechanisms, four RNA-seq libraries were constructed by Z_0_ and Z_1_. These RNA-seq libraries were subjected to pair-end reading with the Illumina HiSeq 4000 platform. In addition, since there is no reference genome sequence in *S. oblata*, all clean reads were de novo assembled into contigs using the Trinity software, and reads were mapped back to contigs, redundancy was removed and the longest transcripts were defined as unigene. The final assembly of *S. oblata* had 73,159 unigenes with an N 50 length of 1116 nucleotides (nt) (Table 2).

**Table 2:** Summary of assembly results of *Syringa oblata* Lindl

Index	All	GC%	Min Length	Median Length	Max Length	Total Assembled Bases	N50
Transcript	162,354	41.20	201	575.00	10,795	134,100,053	1225
Gene	73,159	41.63	201	430	10,795	52,090,718	1116

Unigenes sequence functional annotation.

For functional annotation of the *S. oblata* transcriptome, all assembled unigenes of the 73,159 unigenes were BLASTed against 6 public databases, including NCBI_nr, eggNOG, Swiss-Prot, Pfam, KEGG and GO databases using the DIAMOND [32] program with E-value threshold of 1E-5. From the results in Table 3, a total of 34,764 (42.19%) sequences showed high homology to public databases. Among them, 36,713 (50.18%), 19,592 (26.78%), 32,939 (45.02%), 29,896 (40.86%), 42,229 (57.72%), 42,374 (57.92%) unigenes were annotated in the GO, KEGG, Pfam, Swiss-Prot, eggNOG and Nr databases, respectively.

**Table 3:** Summary of functional annotation of *Syringa oblata* Lindl

DB	Num	Ratio (%)
All	73,159	100.00
GO	36,713	50.18
KEGG	19,592	26.78
Pfam	32,939	45.02
swissprot	29,896	40.86
eggNOG	42,229	57.72
NR	42,374	57.92

Analysis of the Differentially Expressed Genes (DEGs).

The DEGs of the four transcriptome libraries were identified with significant differences expression under the thresholds of log 2 (Fold-change) over 1 and FDR less than 0.001 with an adjusted *p-value* < 0.05. In this study, the expression of genes was calculated by TPM. According to the expression differences, a total of 73,159 genes were detected using KEGG pathway analysis, and only 8015 genes displayed significant changes in expression levels between Z_0_ and Z_1_. The numbers of up-regulated and down-regulated unigenes were 4568 and 3447, respectively in volcano (Additional file 1: Figures S1, S2).

**Fig. 4:**
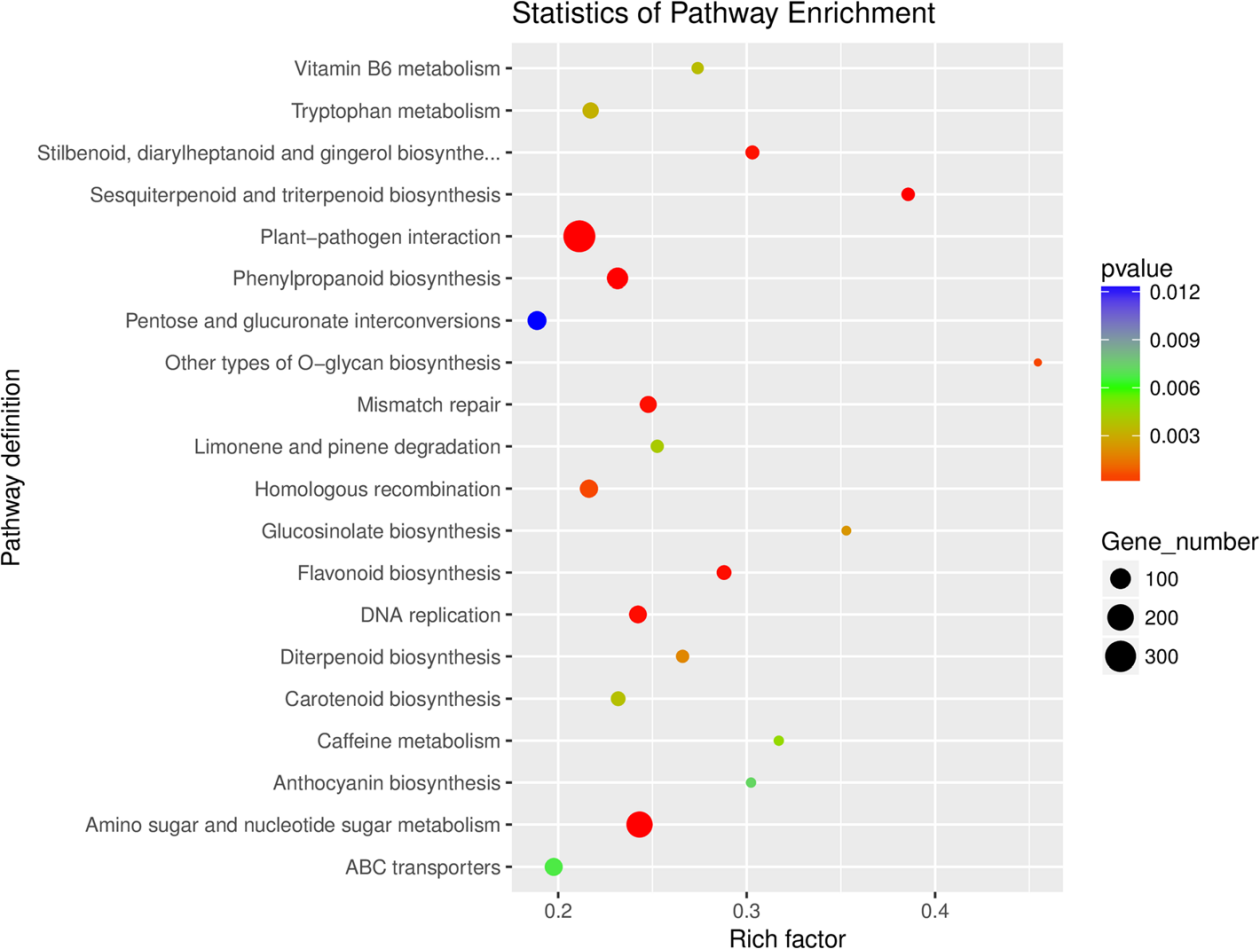
The significantly enriched KEGG pathway of Unigenes in *Syringa oblata* Lindl

GO analysis was performed again based on DEGs in Additional file 1: Figure S3. Of the 36,713 unigenes, 26,072 DEGs were divided into 3 GO terms such as biological process (9588), cellular component (7847) and molecular function (8637). Among them, the most frequently annotated genes involved in the biological process were biological process (627), regulation of transcription, DNA-templated (337) and protein phosphorylation (303). The most frequently annotated genes involved in the cellular component were nucleus (1206), plasma membrane (922) and integral component of membrane (708). And also, the most frequently annotated genes involved in the molecular function were molecular function (607), protein serine/threonine kinase activity (390) and ATP binding (348). The KEGG function annotation were performed and ggplot 2 was used to analyze the KEGG enrichment results as a scatter plot (Fig. 4). The results showed that the top 10 pathways for KEGG enrichment were other types of O-glycan biosynthesis, sesquiterpenoid and triterpenoid biosynthesis, glucosinolate biosynthesis, caffeine metabolism, stilbenoid, diarylheptanoid and gingerol biosynthesis, anthocyanin biosynthesis, flavonoid biosynthesis, vitamin B6 metabolism, diterpenoid biosynthesis and limonene and pinene degradation.

### Integration of metabolites and expressed Unigenes with different light intensity groups

Differential metabolites of flavonoid biosynthesis pathway.

All metabolites related to flavonoid biosynthesis pathway were significantly differentiated by the ratio > 2 or ratio ≤ 1/2, *q-value* ≤ 0.05 and VIP ≥ 1. It included rutin, kaempferol, naringin, tras-2-hydroxycinnamic acid, 3,4-dihydroxyhydrocinnamic acid, astragalin, daidzin, glycitin, L-phenylalanine, N-acetyl-, luteolin, quercetin 3′-methyl ether and so on (Table 4).

**Table 4:** Types of differences in secondary metabolites in the phenylpropanoid pathway

Correlation	ID	RankMS2ident	Ratio	q-value	VIP	Regulated
neg	M609 T415	Rutin	4.49	0.006	1.46	up
neg	M285 T182	Kaempferol	3.41	0.006	1.39	up
neg	M223 T280	Tras-2-Hydroxycinnamic acid	0.45	0.006	1.11	down
neg	M579 T201	Naringin	0.47	0.006	1.06	down
pos	M317 T170	Quercetin 3′-methyl ether	3.20	0.007	1.25	up
pos	M449 T177	Astragalin	3.09	0.021	1.29	up
pos	M287 T178	Luteolin; Kaempferol	3.11	0.007	1.39	up
pos	M381 T271	Daidzin	0.45	0.006	1.09	down
pos	M268 T316	L-Phenylalanine, N-acetyl-	0.32	0.007	1.40	down
pos	M224 T38	3,4-Dihydroxyhydrocinnamic acid	0.40	0.007	1.21	down
pos	M447 T297	Glycitin	0.34	0.021	1.31	down

Differentially expressed Unigenes of flavonoid biosynthesis pathway.

**Fig. 5:**
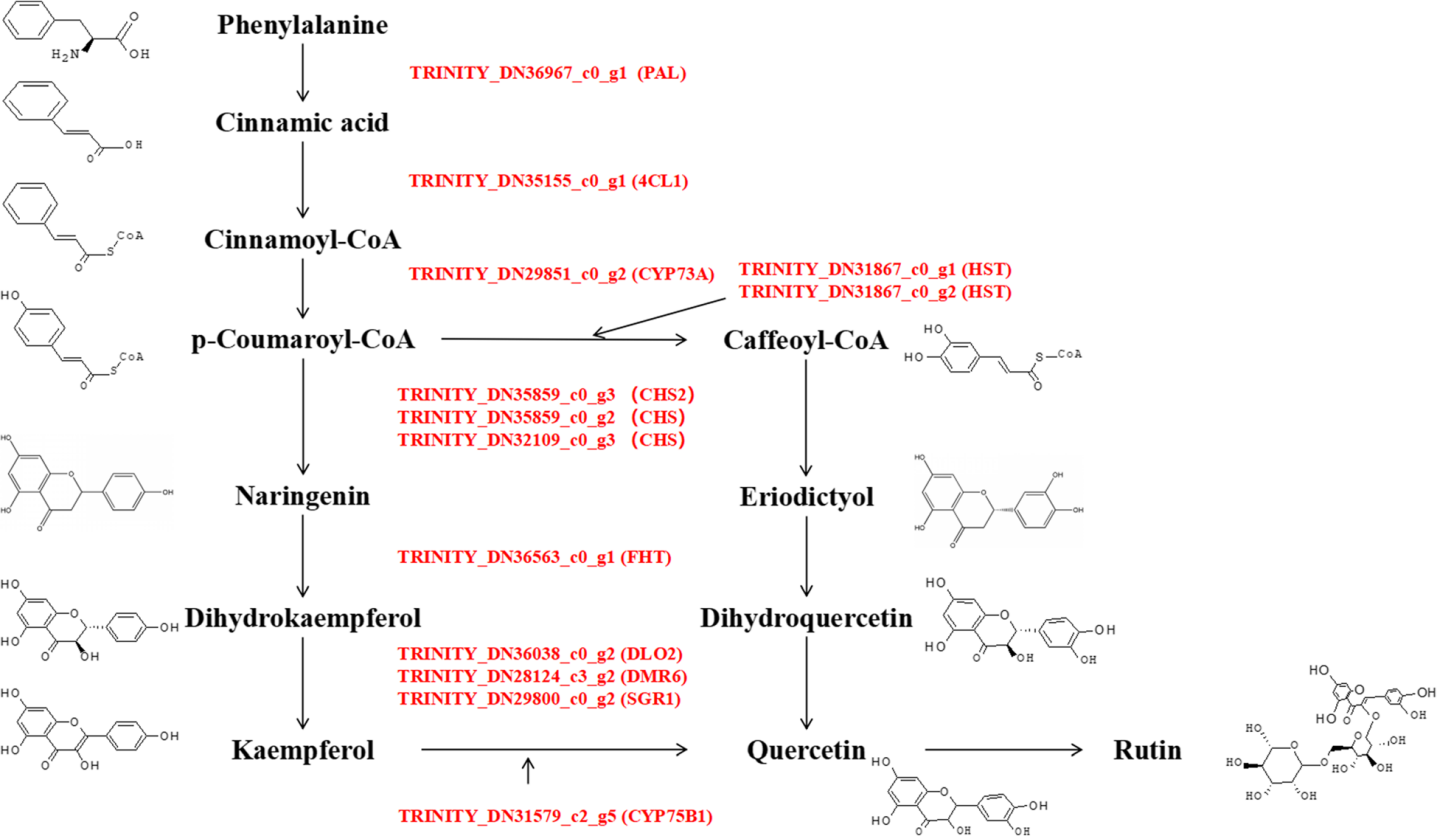
The putative genes involved in flavonoid biosynthesis in *Syringa oblata* Lindl

Based on metabolite results, the content of rutin in *S. oblata* was significantly increased at Z_1_ compared with Z_0_. At the same time, combined with the results of transcriptome sequencing, it was observed that differential metabolite and DEGs were both related to the flavonoid biosynthetic process by GO and KEGG annotation analysis. Thus, based on TPM, 13 putative structural genes and 1 putative regulatory gene involved in the flavonoid biosynthesis pathway were screened as shown in Fig. 5. The results showed that a total of 13 putative structural genes such as phenylalanine ammonia-lyase (*PAL*, TRINITY_DN36967_c0_g1), 4-coumarate-CoA ligase (*4CL1*, TRINITY_DN35155_c0_g1), trans-cinnamate 4-monooxygenase (*CYP73A*, TRINITY_DN29851_c0_g2), two shikimate O-hydroxycinnamoyltransferase (*HST*, TRINITY_DN31867_c0_g1; *HST*, TRINITY_DN31867_c0_g2), three chalcone synthase (*CHS*, TRINITY_DN35859_c0_g3; *CHS*, TRINITY_DN35859_c0_g2; *CHS*, TRINITY_DN32109_c0_g3), naringenin 3-dioxygenase (*FHT*, TRINITY_DN36563_c0_g1), three flavonol synthase (*DLO2*, TRINITY_DN36038_c0_g2; *DMR6*, TRINITY_DN28124_c3_g2; *SGR1*, TRINITY_DN29800_c0_g2), flavonoid 3′-monooxygenase (*CYP75B1*, TRINITY_DN31579_c2_g5), and transcription factor *bHLH* (TRINITY_DN28965_c1_g8) significantly up-regulated the metabolite of rutin in *S. oblata* with Z_1_.

Correlation networks analysis between differential metabolites and differentially expressed Unigenes.

**Fig. 6:**
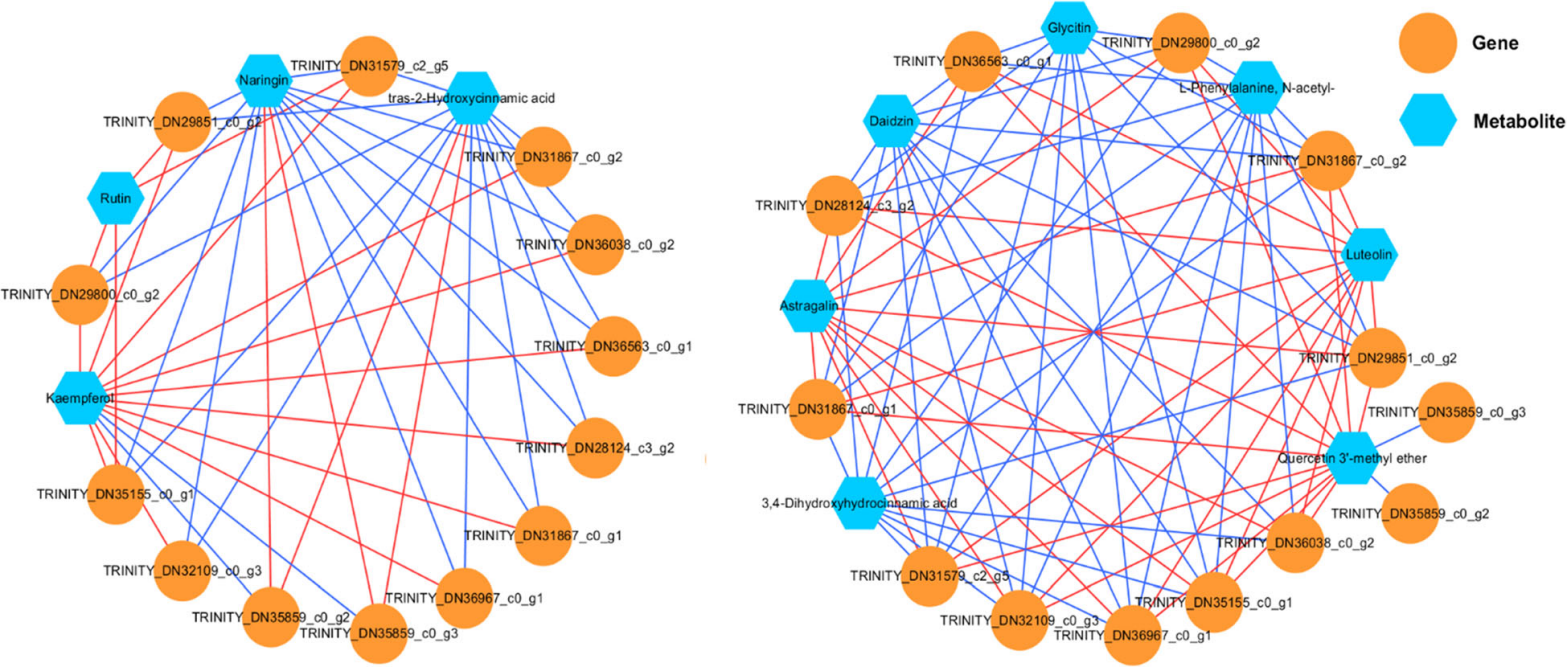
Pearson partial correlation between differential metabolites and expressed Unigenes involved in the biosynthesis of flavonoid in *Syringa oblata* Lindl: left: negative correlation; right: positive correlation

Pearson partial correlation analysis was used to conduct the analysis on the significant correlation networks (r > 0.9 or r < 0.9, *p* < 0.05). The genes and metabolic network properties constructed by 13 putative structural genes and 11 differential metabolites between Z_0_ and Z_1_ could be seen in Fig. 6. The results showed that metabolites of rutin were positively correlated with *4CL1* (TRINITY_DN35155_c0_g1), *CYP73A* (TRINITY_DN29851_c0_g2), *SGR1* (TRINITY_DN29800_c0_g2) and *CYP75B1* (TRINITY_DN31579_c2_g5) under the negative ion mode. These 4 putative genes were negatively correlated and positively correlated with 4 metabolites (rutin, kaempferol, naringin and tras-2-hydroxycinnamic acid) and 7 metabolites (astragalin, daidzin, glycitin, L-phenylalanine, N-acetyl-, luteolin and quercetin 3′-methyl ether), respectively.

Verification of differentially RNA-Seq expressed Unigenes by quantitative real-time PCR.

**Fig. 7:**
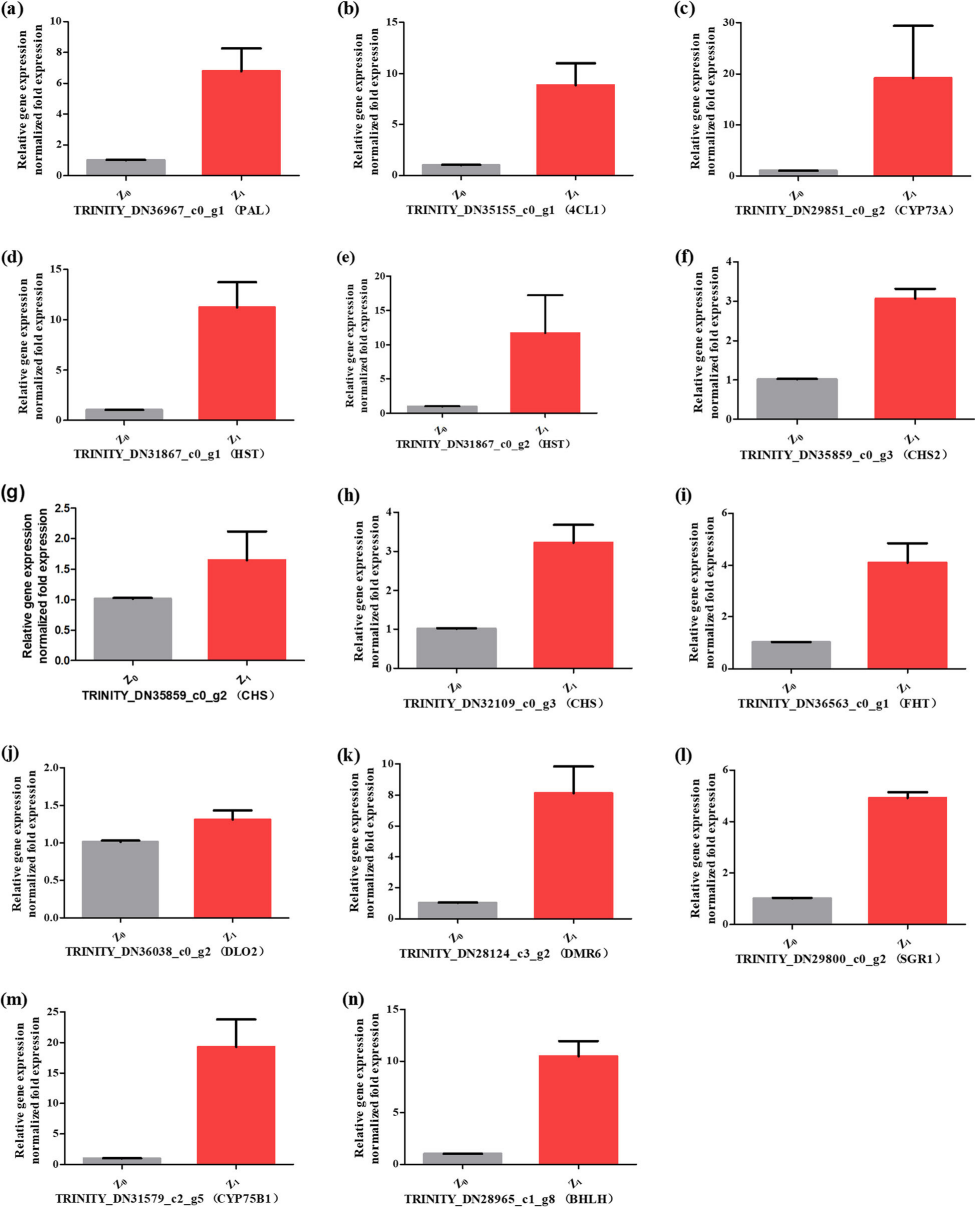
(**a**) *PAL (TRINITY_DN36967_c0_g1)*, (**b**) *4CL1 (TRINITY_DN35155_c0_g1)*, (**c**) *CYP73A (TRINITY_DN29851_c0_g2)*, (**d**) *HST (TRINITY_DN31867_c0_g1)*, (**e**) *HST (TRINITY_DN31867_c0_g2)*, (**f**) *CHS2 (TRINITY_DN35859_c0_g3)*, (**g**) *CHS (TRINITY_DN35859_c0_g2)*, (**h**) *CHS (TRINITY_DN32109_c0_g3)*, (**i**) *FHT (TRINITY_DN36563_c0_g1)*, (**j**) *DLO2 (TRINITY_DN36038_c0_g2)*, (**k**) *DMR6 (TRINITY_DN28124_c3_g2)*, (**l**) *SGR1 (TRINITY_DN29800_c0_g2)*, (**m**) *CYP75B1 (TRINITY_DN31579_c2_g5)*, and (**n**) *bHLH (TRINITY_DN28965_c1_g8)*

To confirm the accuracy of the RNA-Seq sequencing analysis, 13 putative genes were conducted to analyze their relative expression level between Z_0_ and Z_1_ (Fig. 7) by quantitative real-time PCR analysis. The 13 putative genes (*PAL*, *4CL1*, *CYP73A*, two *HST*, two *CHS*, *CHS2*, *FHT*, *DLO2*, *DMR6*, *SGR1*, and *CYP75B1*) expressed from the biosynthesis of rutin were up-regulated and a similar phenomenon was observed with RNA sequencing data, meaning that the transcriptome analysis was reliable. Among them, the putative gene expression of *CYP75B1* (TRINITY_DN31579_c2_g5), *CYP73A* (TRINITY_DN29851_c0_g2), *HST* (TRINITY_DN31867_c0_g2), *HST* (TRINITY_DN31867_c0_g1) and *4CL1* (TRINITY_DN35155_c0_g1) were highest in Z_1_ and up-regulated to 19.31, 19.16, 11.70, 11.23 and 8.84, respectively. This confirms that RNA-Seq sequencing analysis helps to understand the regulatory mechanisms of flavonoid biosynthesis.

## Discussion

*S. xylosus*, one of the most common pathogens in the skin of mammals [33], is frequently isolated from milk, meat, and other food products such as cheeses and sausages [34], it is the leading cause of cow mastitis infection [35]. *S. xylosus* is also one of the coagulase-negative Staphylococci [36], which has strong ability to form biofilm [34, 37]. Therefore, in the quest of eradicating cow mastitis from dairy herds, it is necessary to look for a medicine which can resist the formation of biofilms in bacteria.

Currently, many medicinal plants can inhibit the formation of biofilms, especially among the well-known experiments. Mediterranean herbal extracts have been reported to inhibit the formation of biofilm by *Streptococcus mutans* [38] and Glyeyrrhiza physic liquor, Glabrous crazyweed liquor [39] and Glabrous Crazyweed [40] have also been noted for their activities against the formation of biofilm by *Staphylococcus epidermidis*. This study revealed that 1/2 MIC of *S. oblata* extract in Z_1_ had a significant ability to inhibit *S. xylosus* biofilm formation (*p* < 0.05). At the same time, previous study [41] has confirmed rutin as the main bioactive ingredient in *S. oblata* which inhibits biofilm formation in *S. suis* [15]. This is consistent with the report of this study that rutin is the main bioactive ingredient of *S. oblata* extract which inhibits *S. xylosus* biofilm formation. Therefore, it is significant to improve the content of rutin in *S. oblata* in order to have the best inhibitory effect of *S. oblata* extract against *S. xylosus* biofilm formation.

Current data on PM 2.5 collected from 20 European sites may be pertinent in epidemiologic studies [42]. Visibility degradation is related to the PM components [43]. Light intensity, which is one of the environmental factors in the lower atmosphere, maybe also have effect on the growth of plants, especially in the synthesis of active ingredient and the concentration of their contents [44]. This study revealed that the rutin content of *S. oblata* had a significant increase in August compared to other months (May to October). This finding is contrary to the trend of air pollution index (Additional file 1: Table S1) and earlier findings previously reported on the content of rutin in Fructus Sophorae which was observed to peak in mid-August, and then decline gradually [26]. However, considering the natural growth of *S. oblata* and the role of beautifying the city, it is not recommended to select the month of August as the best harvesting period for *S. oblata*. At the same time, compared with *S. oblata* extract in August, there was no significant difference in the biofilm formation of *S. xylosus*. Therefore, it is recommended that the harvesting period of *S. oblata* should be selected before the emergence of the dry leaves in mid-September, which is consistent with previous studies [26]. In addition, previous study has found that anthocyanin and flavonoids accumulation is strongly associated with different flowering developmental stages in *S. oblata* [45, 46]. With regard to photoperiod, it was observed that flavonol compounds and the expression of flavonoid pathway genes were related to the increased light exposure imposed on sweet potato leaves [47]. In summary, this is consistent with this study that the amount of rutin content was related to the plant photoperiod or developmental stage. Shading treatments significantly affected flavonoid accumulation in plants, such as in tea plants [48]. However, the flavonoid levels in an excellent albino tea germplasm increased after moderate shading treatment [49] and all fruits do not require strong light exposure to accumulate high amounts of flavonoids [50]. And the shaded trees were significantly lower than trees without shades (exposed to sun) to enhance light capture and use efficiency in low-light environments [51], but would not greatly alter spectral quality [52]. This is consistent with this study that the PAR of Z_1_ and Z_2_ were significantly lower than Z_0_ (Fig. 2b). The light radiation of Z_1_ were significantly lower than Z_0_ group (Fig. 2c), which has a lot of factors related to the results such as the cloud cover in the sky and the observation angles, but the spectrum is basically the same. Thus, this study further confirmed that 1/2 MIC of *S. oblata* extract had a significant ability to inhibit *S. xylosus* biofilm formation (*p* < 0.05) and rutin content in *S. oblata* increased significantly under Z_1_ compared with Z_0_ in September 2017. So, in this study, the results about the increasing of flavonoid content in lower light intensities for *S. oblata* is not only unique to *S. oblata*. This indicated that the selection of *S. oblata* in Z_1_ is of great significance for subsequent molecular mechanism research because of its excellent ability to inhibit biofilm formation and the high rutin content compared with Z_0_.

Furthermore, in order to locate the subcellular site of flavonoids accumulation in the tissues, histochemical analysis was conducted. Previous studies have shown that the key enzyme in flavonoids formation was found in the epidermal [53] and subepidermal mesophyll tissue that could absorb potentially harmful UV-B radiation [28]. The results showed that more accumulation of flavonoids was observed in Z_1_ in the epidermal cell layers and vascular bundles compared with Z_0_. This finding is in agreement with earlier findings previously reported [28]. Generally, the study on the accumulation of flavonoids in *S. oblata* under Z_0_ and Z_1_ was used to investigate the molecular mechanism that was beneficial to flavonoid production.

Up to date, metabolome and transcriptomic data of *S. oblata* are still not available in NCBI database, which is widely used in identifying novel genes that are involved in the biosynthesis of secondary metabolites. Thus, a metabolite and RNA-sequencing analysis without a reference genome was used to elucidate the differential regulation involved in the different light intensity groups for *S. oblata*. In the current study, the results indicated that the differential metabolites and DEGs which were annotated and classified were mainly related to the flavonoid biosynthesis pathway. It has been reported that the components involved in flavonoid biosynthesis are the main functional components in many species. For example, the flavonoid biosynthesis related genes in *Tricyrtis sp* were isolated and characterized and they consist of *CHI*, *F3H*, *F3’H*, *FLS*, *DFR*, *ANS*. These genes vary with the flower developmental stages [45]. 16 genes were different in the regulation of flavonoid biosynthesis in *Camellia sinensis* under different shading stages. It was observed that *F3’H* and *FLS* significantly decreased throughout the shading stages while the others (*PAL*, *CHS*, *DFR*, *ANS*, *ANR* and *LAR*, etc.) temporally decreased in the early or late shading stages [48]. Interestingly, compared with Z_0_, this study showed that 13 putative structural genes related to the flavonoid pathway were up-regulated in Z_1_, indicating that these putative genes may be the key target genes regulating flavonoid biosynthesis and metabolism.

Furthermore, 3 putative genes including *4CL1* (TRINITY_DN35155_c0_g1), *CYP73A* (TRINITY_DN29851_c0_g2) and *CYP75B1* (TRINITY_DN31579_c2_g5) were positively correlated with rutin by the integration of metabolites and DEGs analysis and up-regulated to 19.31, 19.16, 11.70, 11.23 and 8.84 respectively by quantitative real-time PCR analysis. This suggests that our results provide the first accurate and relevant gene information for *S. oblata* in the flavonoid biosynthetic pathway.

*4CL1* is the first main branch point enzyme that controls the metabolism of rutin through the phenylpropanoid metabolic pathway. *4CL* enzymes catalyzes the conversion of several hydroxycinnamic acids into their corresponding thioesters, conserving the evolution of vascular plants [54]. In addition, the *4CL* enzymes have been characterized from various plants for their role in plant physiology or in biotic and abiotic stresses [55]. In this study, *4CL1* (TRINITY_DN35155_c0_g1) was positively correlated with Z_1_ which helped to promote most of the enzymes including *CYP73A* (TRINITY_DN29851_c0_g2) and *CYP75B1* (TRINITY_DN31579_c2_g5). In addition, de novo transcriptome sequencing was performed to reveal that genes such as curcumin synthase and *CYP73A* were the differentially expressed genes in the different species of gingers being investigated [56]. At the same time, expression of chalcone reductase, flavonoid 3′,5′-hydroxylase (*F3’5’H*) and *CYP75B1* were all reported to be up-regulated under cold treatment in *C. microphyllum*, which was as a potential source of abiotic stress resistant germplasm for chickpea breeding programs [57]. This study has confirmed that the presence of *CYP73A* (TRINITY_DN29851_c0_g2) and *CYP75B1* (TRINITY_DN31579_c2_g5) enhanced promote the production of rutin.

In addition, studies have shown that transcriptional regulation such as *bHLH* is the most important tool for modulating flavonoid biosynthesis when plants are under stress conditions [58]. In plants, *bHLH* transcription factors have a wide range of functions, such as regulation of marginal pollen tract tissues growth [59], photomorphogenesis [60] plant growth, development and defence [61] and flavonoid biosynthesis [62]. The study observed that three *bHLH* (*bHLH79*, *bHLH147*, and *bHLH79*-like) transcription factors were up-regulated at the budding and flowering stages, and another two transcription factors (*bHLH3*, and *bHLH48*) were down-regulated at the budding and flowering stages in *S. oblata* [46]. The over-expression of *bHLH1* gene from grape was chemically synthesized and significantly increased the accumulation of flavonoids and enhanced salt and drought tolerance in transgenic *Arabidopsis thaliana* plants [63]. This experiment showed that under light intensity groups, the *bHLH* transcription factor was up-regulated to 10.48. At the same time, it was observed to be the main factor that regulated the flavonoid biosynthetic process, seed coat development, protein dimerization activity, seed development, regulation of proanthocyanidin biosynthetic process and so on.

## Conclusions

The synthesis of the active ingredient content of *S. oblata* will be affected under different light intensity groups. Therefore, it was speculated that a smoggy environment would affect the synthesis of the secondary metabolic components in medicinal plants. Based on our work view, we successfully screened *S. oblata* under Z_1_ and it was observed to have the best rutin and flavonoids content and a better efficacy in inhibiting *S. xylosus* bioflm formation. The result of metabolite and transcriptome analysis revealed significant metabolite-genes that correlated with the flavonoids biosynthesis pathways such as *4CL1* (TRINITY_DN35155_c0_g1), *CYP73A* (TRINITY_DN29851_c0_g2) and *CYP75B1* (TRINITY_DN31579_c2_g5), and therefore present a better understanding of the molecular mechanism behind the accumulation of flavonoids by *S. oblata* in response to different light intensity.

## Materials and methods

### Plant growth and materials collection

*S. oblata*, identified by Professor Xiuju Wu (College of Life Sciences, Northeast Agricultural University, Harbin, China) were grown under natural sunlight environment in the campus of Northeast Agricultural University (N 45°44′33.64″, E 126°43′22.07″) in Harbin, Heilongjiang Province of China. They were grown under natural environmental conditions without additionally watered/fertilized. The experiment was divided into two groups of different months and different light intensity groups. The first batch of experimental samples were collected from the *S. oblata* grouped under different months and sample collection started on the 11th of May and ended on 11th of October, 2017. The other experiment was conducted by using different black coloured nets to cover the *S. oblata* and thus different light intensity treatments were created as follows: *S. oblata* under natural growth condition (Z_0_), *S. oblata* with 40% ~ 50% shading treatment (Z_1_, 50% ~ 60% of natural sunlight can be transmitted through the shade nets) and *S. oblata* with 65% ~ 75% shading treatment (Z_2_, 25% ~ 35% of natural sunlight can be transmitted) (Fig. 1a and Fig. 2a). The nets were placed over the plants on 11th of May in 2017 (Fig. 2a). *S. oblata* leaves were collected from all the different light intensity treatments on 11th of September in 2017. At the same time, environmental parameters were measured among each treatment to monitor the growth conditions of *S. oblata* during light intensity experiment, including the PAR (Light Scout® Quantum Light Meters, Item#3415F, Spectrum Technology® Inc. USA) and light reflection by spectrometer (UVCHR768, SVC, America). Above all, in each of the experiment, five trees were selected from each treatment group, and a large number of leaves were collected from each tree randomly [51]. All samples were collected on the 11th of each month, frozen in liquid nitrogen and stored at − 80 °C until they were analyzed.

### Biofilm formation ability of *S. xylosus*

*S. xylosus* ATCC 700404 strain was cultured in Trypticase Soy Broth (TSB: Summus Ltd., Harbin, Heilongjiang, China) at 37 °C for 12 h with constant shaking. The methanolic extracts of *S. oblata* and standard rutin were used for MIC assays using the protocol described previously [64]. Briefly, the overnight cultures of *S. xylosus* were diluted to a density of 1 × 10^5^ CFU/mL using sterile TSB, then 100 μL samples were added to each well in a 96-well plate (Corning Costar® 3599 Corning, NY, USA) containing serial dilutions of compounds in 100 μL culture medium. Control bacteria were cultivated in the absence of extracts of *S. oblata*. The MICs were determined as the lowest concentration of extracts of *S. oblata* after incubation for 24 h at 37 °C. The biofilm formation assay was carried out by the extracts of *S. oblata* using 96-well microtiter plates [64]. Negative control wells contained broth only. Positive control wells contained culture medium and bacterial suspension. Biofilms were treated as described above [64]. The OD of sample was measured at 595 nm using a micro-titer plate reader (DG5033A, Huadong Ltd., Nanjing, Jiangsu, China). Three independent sample analysis were performed for each tissue.

### Determination of rutin content

High performance liquid chromatography (HPLC) analysis was performed on methanolic extracts of *S. oblata* using Waters Alliance HPLC system (Shimadzu, Corporation, Kyoto, Japan) that is equipped with a binary pump and a UV/V detector. The fresh tissues obtained from the different months and light intensity groups which were frozen in liquid nitrogen were dried under room temperature. A total of 20 to 50 mg for each dry sample was used to extract flavonoids by adding 2 mL of 50% (*v/v*) of methanol (HPLC grade) in H_2_O. Then, the mixture was placed in an ultrasonic cleanser (Ningbo Scientz Biotechnology Co. Ltd) for 20 min and centrifuged for 10 min at 13000 rpm [9]. The supernatant was filtered through a 0.45 mm membrane filter and loaded for HPLC analysis. The chromatographic separation was carried out on a Diamosil C18 column (4.6 mm × 250 mm, 5 μm) with a gradient solvent A (0.1% formic acid aqueous solution) and solvent B (acetonitrile) as mobile phase at a flow rate of 1 mL/min. The gradient conditions was 0 min, 5% solvent B; 30 min, 53% solvent B; 35 min, 5% solvent B [15]. Rutin quantity was estimated based on the linear calibration curve of standard rutin (Sigma-Aldrich, Germany) under a detection wavelength of 355 nm. Three independent sample analysis were performed for each tissue.

### Histochemical analysis of flavonoids with different light intensity groups

Small pieces of fresh *S. oblata* leaves (Z_0_ and Z_1_) were embedded in medium before cutting for histolocalisation as described above [65, 66]. The 20-μm embedded tissue were obtained using a LEICA CM 1850P vibrating blade microtome. These sections were labeled with saturated (0.25%, *w/v*) 2-aminoethyl diphenylborinate (DPBA) (Macklin, Shanghai Macklin Biochemical Co.,Ltd.) that dissolved in 80% methanol for 15 min. Then, 80% methanol was used to wash away the excess DPBA dye and xylene was used to make the tissues transparent. The DPBA-labelled sections were viewed by laser confocal scanning microscopy (LCSM, Germany).

### Metabolites extraction and LC-MS analysis with different light intensity groups

#### Metabolites Extraction

Frozen samples from Z_0_ and Z_1_ were ground into fine powder in liquid nitrogen. One hundred milligrams of powder from each sample was extracted with 120 μL of precooled 50% methanol, vortexed for 1 min, and incubated at room temperature for 10 min. Then, the extraction mixture was stored overnight at − 20 °C. After centrifugation at 4000 g for 20 min, the supernatants were stored at − 80 °C prior to the LC-MS analysis. Six biological repeats were performed and both data sets produced qualitatively similar results.

#### LC-MS analysis

Metabolite analysis was performed using an ultra-performance liquid chromatography (UPLC) system (SCIEX, UK). An ACQUITY UPLC BEH Amide column (100 mm × 2.1 mm, 1.7 μm, Waters, UK) was used for the reversed phase separation, a high-resolution tandem mass spectrometer TripleTOF5600 plus (SCIEX, UK) was used to detect the metabolites eluted from the column. At the last, the specific instrument parameters and conditions were set up using settings as previously described [67].

#### Metabolite data analysis

LC-MS raw data files were converted into mzXML format and then processed by the XCMS (UC, Berkeley, CA, USA) [68], CAMERA [69] and metaX toolbox [70] implemented with the R software. Each ion was identified by combining retention time (RT) and m/z data. In order to explain the physical, chemical properties and biological functions of metabolites, the online Kyoto Encyclopedia of Genes and Genomes (KEGG) (http://www.kegg.jp/) databases were used to perform identification and annotation. Screening and quantitative analysis for differential metabolites were conducted using metaX software (http://metax.genomics.cn/) [67]. At the same time, the normalized data was then used to perform PCA and PLS-DA [71]. Wilcoxon tests were conducted to detect differences in metabolite concentrations between 2 phenotype. The *P* value was adjusted for multiple tests using an FDR (Benjamini - Hochberg). The PLS-DA was conducted through metaX to determine the differences in the different variables between groups. Then VIP value was calculated and also a VIP cut-off value of 1.0 was used to select important features [67].

### Transcriptome sequencing and data analysis with different light intensity groups

#### RNA extraction

About 100 mg fresh sample from Z_0_ and Z_1_ were ground into fine powder in liquid nitrogen and then stored at − 80 °C for RNA extraction. Total RNA was extracted using Trizol reagent (Invitrogen, CA, USA) by following the manufacturer’s procedure. The total RNA quantity and purity were analyzed by Bioanalyzer 2100. Two biological repeats were performed.

#### RNA library construction and transcriptomic analysis

Approximately 10 μg of total RNA were prepared. The methods adopted for library preparation, de novo strategy and transcriptomic analysis were the same with our previous study [72]. Sequencing was carried out using an Illumina Hiseq 4000 platform (LC-Bio, Hangzhou, China) according to the manufacturer’s protocol. De novo assembly of the transcriptome was performed with Trinity 2.4.0 [73]. For gene identification and expression analysis, the reads from different species were co-assembled, and for gene sequence analysis, the reads from different species were assembled separately.

#### Unigenes annotation and functional classification

All assembled Unigenes were aligned against the non-redundant (Nr) protein database (http://www.ncbi.nlm.nih.gov/), Gene ontology (GO) (http://www.geneontology.org), SwissProt (http://www.expasy.ch/sprot/), KEGG (http://www.genome.jp/kegg/) and eggNOG (http://eggnogdb.embl.de/) databases using DIAMOND [32] with a threshold of E-value< 0.00001.

#### Differentially expressed Unigenes analysis

Salmon [74] was used to calculate the expression level of Unigenes (TPM) [75]. The differentially expressed Unigenes (DEGs) were selected with log 2 (fold change) > 1 or log 2 (fold change) < − 1 and with statistical significance (*p value* < 0.05) by R package edgeR [76]. Next, GO and KEGG enrichment analysis were again performed on DEGs by perl scripts in-house.

### Integration of metabolites and expressed Unigenes with different light intensity groups

#### Functional analysis of integration of metabolomics and transcriptomics data

Correlation between the expression levels of 13 putative genes and the profiles of flavonoids in *S. oblata* from Z_0_ and Z_1_ were carried out using the program R 2.10.1. RPKM values for genes and the peak values of metabolites were used as a matrix for pearson partial correlation analysis [77]. The metabolite correlation network was constructed for Z_0_ and Z_1_ using all metabolite accumulation profiles separately. Correlation pairs were deemed statistically significant when the |PCC| > 0.9 and *p*-value < 0.01. The resulting correlation networks were obtained and used for network visualization and analysis of network properties using Cytoscape software (Cytoscape 2.6.3) [78].

### Verification of differentially expressed Unigenes in flavonoid biosynthesis pathway by quantitative real-time PCR

The total RNA for quantitative real-time PCR analysis was extracted using TRIZOL reagent and 1.0 μg RNA was used for reverse transcription using the PrimeScript™ RT reagent Kit with gDNA Eraser (Tiangen, Beijing, China) in 20 μL of reacting system. 13 putative structural genes and 1 putative regulatory gene were selected as DEGs. The CPR gene were selected as internal control. The specific primers were designed from Sangon Biotech (Shanghai) and listed in Additional file 1: Table S2. Quantitative real-time PCR was performed using the Roche Light Cycle 480 II sequence detection system (Roche, Switzerland) as previously described, with a few modifications [79]. The final volume for each reaction was 10 μL with the following components: 1 μL diluted cDNA template (1 mg/mL), 5 μL SYBR Green Master (ROX) (Indianapolis, IN, USA), 0.3 μL forward primer, 0.3 μL reverse primer and 3.4 μL ddH_2_O. The reaction was set at 95 °C for 10 min, followed by 40 cycles of denaturation at 95 °C for 15 s and annealing/extension at 60 °C for 1 min. Each quantitative real-time PCR analysis was performed with three biological replicates.

### Student’s t-test

Values were expressed as means ± SDs. The statistical differences among different groups were compared by 1-way ANOVA. Significant means were separated using Tukey method and statistical significant level was set as *p* < 0.05. The data of quantitative real-time PCR were analyzed using repeated measurements in -ΔCt model [80].

## Supplementary information

**Additional file 1: Figure S1.** Genes with significant differential expression in *Syringa oblata* Lindl. **Figure S2.** Volcano diagram of differentially expressed Unigenes between two groups in *Syringa oblata* Lindl. **Figure S3.** GO enrichment analysis of differentially expressed Unigenes in *Syringa oblata* Lindl. **Table S1.** Air pollution index for May to October in 2017. **Table S2** Fluorescence quantitative real-time PCR primer sequence.

## Data Availability

All raw sequence reads have been deposited in NCBI’s Gene Expression Omnibus and are accessible through GEO Series accession number GSE137862 (https://www.ncbi.nlm.nih.gov/geo/query/acc.cgi?acc=GSE137862).

## Abbreviations

*4CL1*: 4-coumarate-CoA ligase
*CHS*: chalcone synthase
*CYP73A*: trans-cinnamate 4-monooxygenase
*CYP75B1*: flavonoid 3′-monooxygenase
DEGs: Differentially Expressed Genes
*DLO2*, *DMR6* and *SGR1*: flavonol synthase
DPBA: 2-aminoethyl diphenylborinate
*F3’5’H*: flavonoid 3′,5′-hydroxylase
*FHT*: naringenin 3-dioxygenase
GO: Gene ontology
HPLC: High performance liquid chromatography
*HST*: shikimate O-hydroxycinnamoyltransferase
KEGG: Kyoto Encyclopedia of Genes and Genomes
MIC: minimal inhibitory concentration
*PAL*: phenylalanine ammonia-lyase
PCA: principal component analysis
PLS-DA: partial least squares-discriminate analysis
PM: particulate matter
*S. oblata*: *Syringa oblata* Lindl
*S. Suis*: *Streptococcus suis*
*S. Xylosus*: *Staphylococcus xylosus*
