# Supplementary material for: Transcriptomic analysis reveals flavonoid biosynthesis of Syringa oblata Lindl. in response to different light intensity
Source: BMC Plant Biol. 2019 Nov 11;19:487. doi: 10.1186/s12870-019-2100-8 (PMC6849326; doi:10.1186/s12870-019-2100-8)
Supplement: Supplementary file 1 — Additional file 1: Figure S1. Genes with significant differential expression in Syringa oblata Lindl. Figure S2. Volcano diagram of differentially expressed Unigenes between two groups in Syringa oblata Lindl. Figure S3. GO enrichment analysis of differentially expressed Unigenes in Syringa oblata Lindl. Table S1. Air pollution index for May to October in 2017. Table S2 Fluorescence quantitative real-time PCR primer sequence. [file 12870_2019_2100_MOESM1_ESM.docx]

**Transcriptomic Analysis Reveals Flavonoid Biosynthesis of *Syringa oblata* Lindl.** **in Response to Different Light** **Intensity**


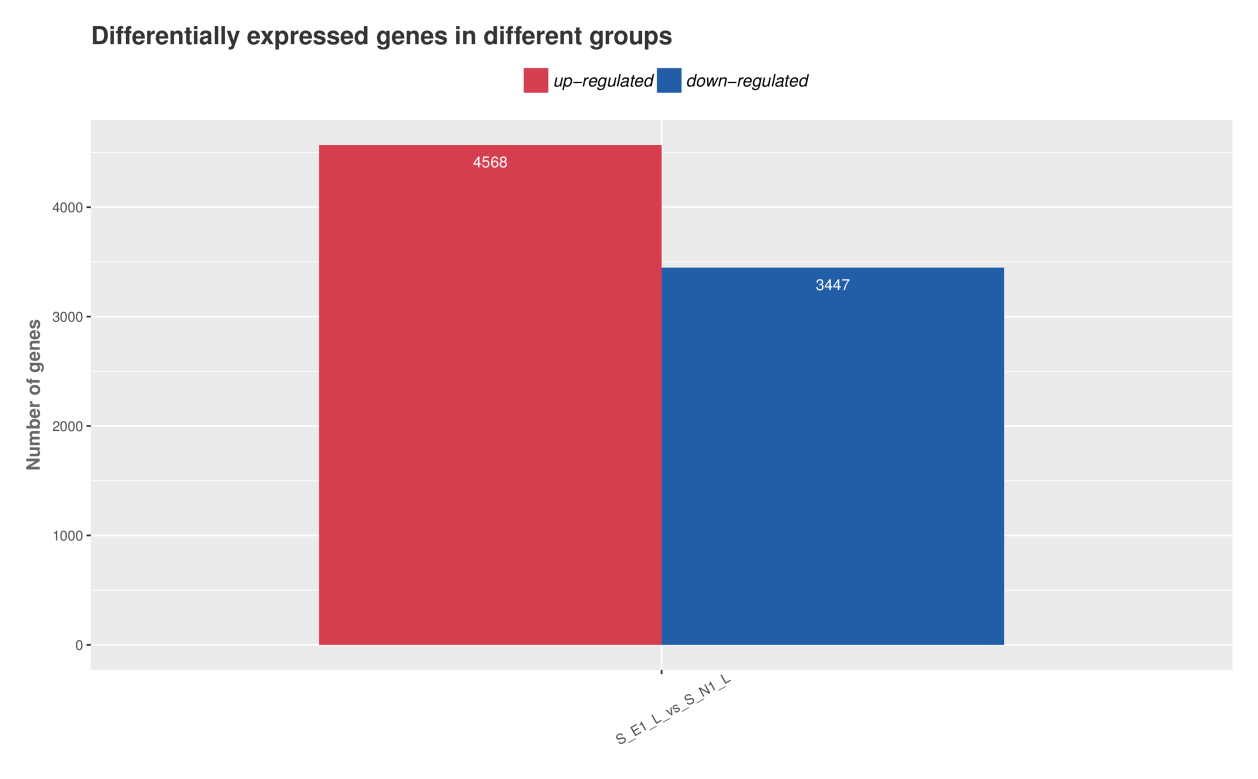
**Figure S1** Genes with significant differential expression in *Syringa oblata* Lindl.


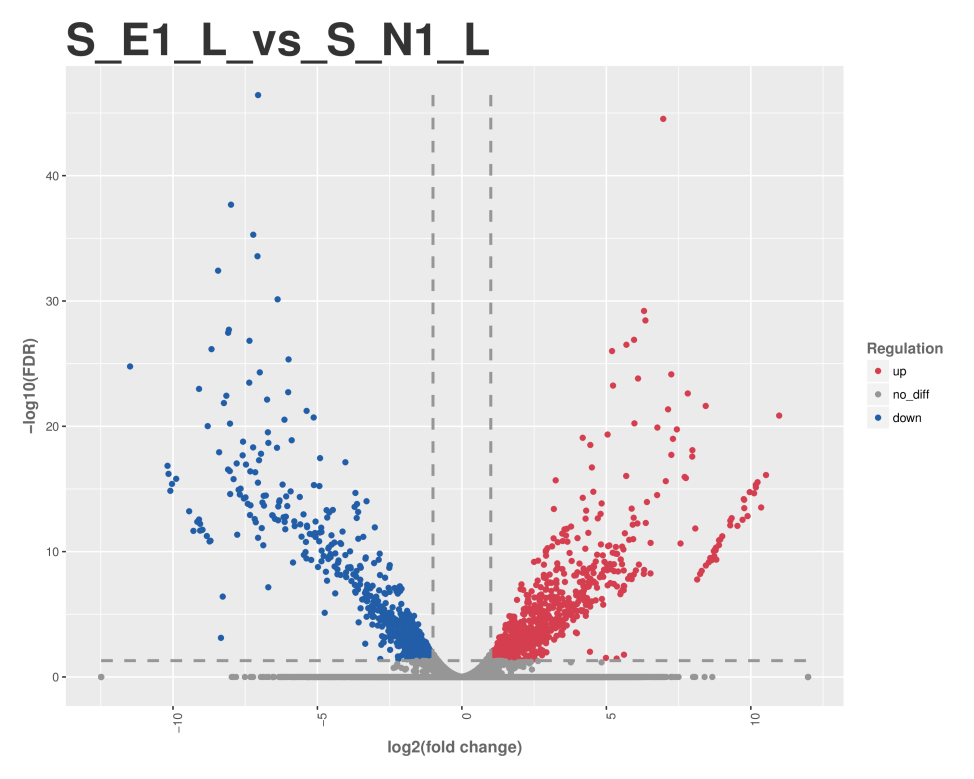


**Figure S2** Volcano diagram of differentially expressed Unigenes between two groups in *Syringa oblata* Lindl.


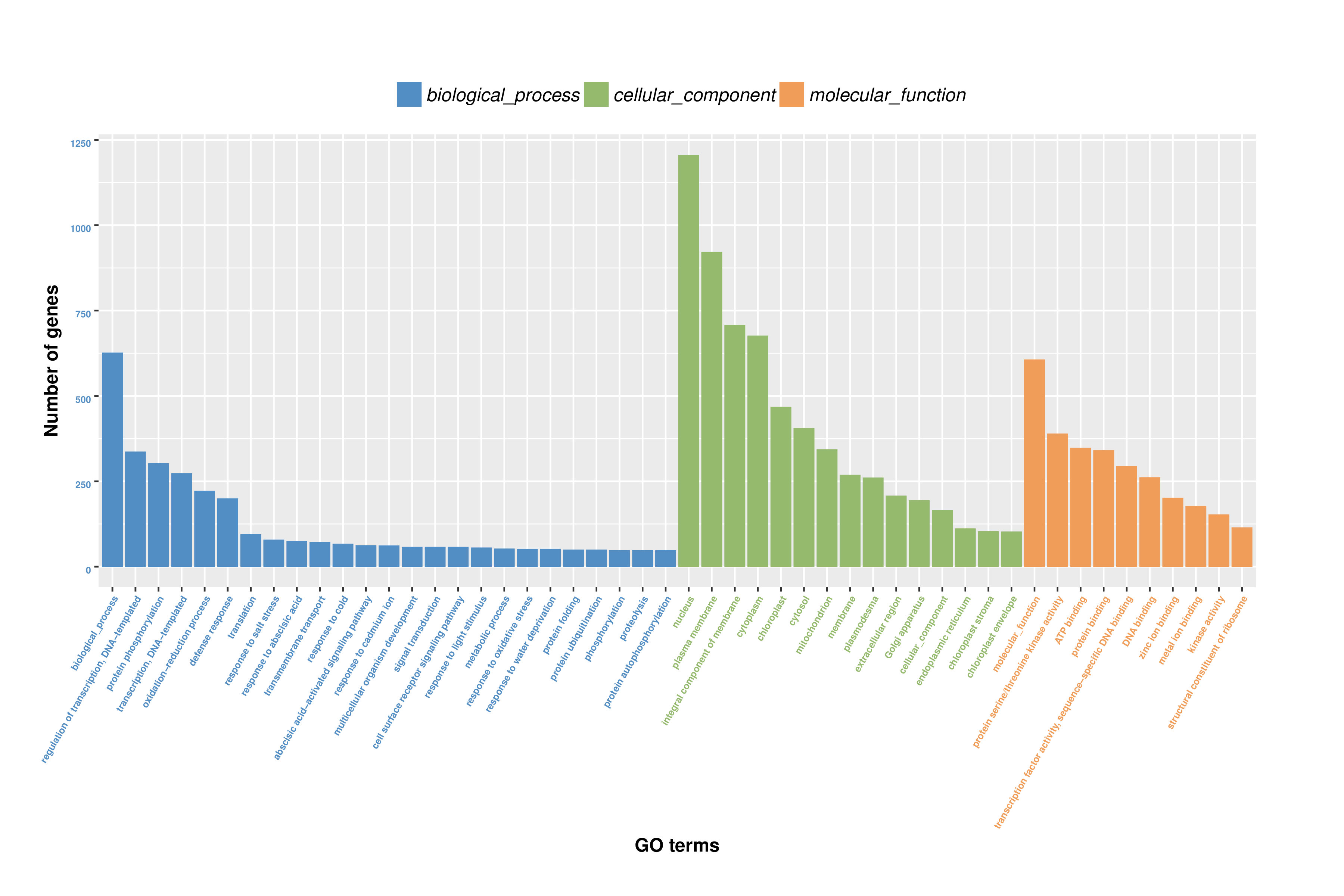


**Figure S3** GO enrichment analysis of differentially expressed Unigenes in *Syringa oblata* Lindl.

**Table S1** Air pollution index for May to October in 2017.

| Month | AQI | Range | Quality level | PM  2.5 | PM  10 | SO_2_ | CO | NO_2_ | O_3_ |
| --- | --- | --- | --- | --- | --- | --- | --- | --- | --- |
| May | 85 | 37~297 | Good | 24 | 65 | 11 | 0.877 | 31 | 122 |
| June | 68 | 32~148 | Good | 24 | 45 | 9 | 0.723 | 37 | 114 |
| July | 74 | 42~128 | Good | 21 | 50 | 8 | 0.713 | 37 | 124 |
| August | 48 | 27~85 | Excellent | 16 | 38 | 7 | 0.574 | 28 | 88 |
| September | 48 | 29~70 | Excellent | 21 | 48 | 8 | 0.637 | 33 | 74 |
| October | 119 | 35~473 | Mild pollution | 94 | 122 | 17 | 0.848 | 49 | 84 |

**Table S2** Fluorescence quantitative real-time PCR primer sequence.

| Gene_ID | Primer | Primer sequence（5' - 3'） |
| --- | --- | --- |
| TRINITY_DN36967_c0_g1 (PAL) | Forward | AGATTGAGGCTGCTGCTGTGATG |
|  | Reverse | CGATCCTGCTTCGGCTTCTGC |
| TRINITY_DN35155_c0_g1 (4CL1) | Forward | GGACGCAGGAGAAGTTCCAGTTG |
|  | Reverse | CGACGGAGACTTAGGAATGGCATC |
| TRINITY_DN29851_c0_g2 (CYP73A) | Forward | CGCCGCCTCTGTTCCATTCTC |
|  | Reverse | ATAATCCGGCATGGTGGAAGAAGC |
| TRINITY_DN31867_c0_g1 (HST) | Forward | CATGGTCTGATATGGCTCGTGGTC |
|  | Reverse | CGGCTGGTACTCGATGTGCTTG |
| TRINITY_DN31867_c0_g2 (HST) | Forward | TTCTAGGCTCCAACCTCCACTTCC |
|  | Reverse | GGAGATCACCAGCTACTGCGATTG |
| TRINITY_DN35859_c0_g3 (CHS2) | Forward | TGGTTACGGTTGAGGATGTTCGTC |
|  | Reverse | CAATTCGGTGGCGTTGCAGTG |
| TRINITY_DN35859_c0_g2 (CHS) | Forward | GCAGAGCACCACAGGCGAAG |
|  | Reverse | CAGAACCACGGTCTCAACTGTCAG |
| TRINITY_DN32109_c0_g3 (CHS) | Forward | GCCGACTACCAACTCACCAAGC |
|  | Reverse | TGTTGTTCTCCGCCAAGTCCTTG |
| TRINITY_DN36563_c0_g1 (FHT) | Forward | GATGACGATGACGGCGGCAAG |
|  | Reverse | TGTCGAACCGGAGCTTCTCTATGG |
| TRINITY_DN36038_c0_g2 (DLO2) | Forward | GGACGCTGGTGCCTTGACAATC |
|  | Reverse | CTCGGAATTGACCTTCGCTCTGTG |
| TRINITY_DN28124_c3_g2 (DMR6) | Forward | AACTTATGGTCTTCCGGCTCATGC |
|  | Reverse | TGCCATCCTTGAGAACTTGCAGAC |
| TRINITY_DN29800_c0_g2 (SGR1) | Forward | CGCCTCTTCCACCCAAGAAC |
|  | Reverse | GAGGCTGTGCCACCAATTCT |
| TRINITY_DN31579_c2_g5 (CYP75B1) | Forward | ACATGCATACGACTTACCGTGG |
|  | Reverse | GCGGGTGGAGATTGGTCTATG |
| TRINITY_DN28965_c1_g8 (BHLH) | Forward | GGAAGACAGTTCAGCCGACAGAAG |
|  | Reverse | GGCGACAAGGCAGCAGAAGG |
| TRINITY_DN36649_c0_g3 (CPR) | Forward | GTCCAACACAAGATGGCTGAGAGG |
|  | Reverse | AGCCTTGGTGCTGTCTAGAGATCC |
